# Resveratrol, but not EGCG, in the diet suppresses DMBA-induced mammary cancer in rats

**DOI:** 10.1186/1477-3163-5-15

**Published:** 2006-05-15

**Authors:** Timothy Whitsett, Mark Carpenter, Coral A Lamartiniere

**Affiliations:** 1 Department of Pharmacology and Toxicology, University of Alabama at Birmingham, Birmingham, AL, USA; 2 Comprehensive Cancer Center, University of Alabama at Birmingham, Birmingham, AL, USA; 3 Department of Mathematics and Statistics, Auburn University, Auburn, AL, USA

## Abstract

Despite the advent of new and aggressive therapeutics, breast cancer remains a leading killer among women; hence there is a need for the prevention of this disease. Several naturally occurring polyphenols have received much attention for their health benefits, including anti-carcinogenic properties. Two of these are resveratrol, a component of red grapes, and epigallocatechin-3-gallate (EGCG), the major catechin found in green tea. In this study, we tested the hypothesis that these two polyphenols protect against chemically-induced mammary cancer by modulating mammary gland architecture, cell proliferation, and apoptosis. Female Sprague-Dawley CD rats were exposed to either resveratrol (1 g/kg AIN-76A diet), EGCG (0.065% in the drinking water), or control diet (AIN-76A) for the entirety of their life starting at birth. At 50 days postpartum, rats were treated with 60 mg dimethylbenz[a]anthracene (DMBA)/kg body weight to induce mammary cancer. Resveratrol, but not EGCG, suppressed mammary carcinogenesis (fewer tumors per rat and longer tumor latency). Analysis of mammary whole mounts from 50-day-old rats revealed that resveratrol, but not EGCG, treatment resulted in more differentiated lobular structures. Bromodeoxyuridine (BrdU) incorporation studies showed that resveratrol treatment caused a significant reduction in proliferative cells in mammary terminal ductal structures at 50 days postpartum, making them less susceptible to carcinogen insult. The epithelial cells of terminal end buds in the mammary glands of resveratrol-treated rats also showed an increase in apoptotic cells compared to the control or EGCG-treated rats as measured by a DNA fragmentation assay. At the given doses, resveratrol treatment resulted in a serum resveratrol concentration of 2.00 μM, while treatment with EGCG resulted in a serum EGCG concentration of 31.06 nM. 17β-Estradiol, progesterone, and prolactin concentrations in the serum were not significantly affected by resveratrol or EGCG. Neither polyphenol treatment resulted in toxicity as tested by alterations in body weights, diet and drink consumptions, and day to vaginal opening. We conclude that resveratrol in the diet can reduce susceptibility to mammary cancer, while EGCG in the drinking water at the dose used was not effective.

## Background

Breast cancer remains a leading killer among cancers that affect women in the United States and around the world. It was estimated that in 2005, in the US alone, there were 211,240 new cases of female breast cancer and 40,410 deaths [1]. This remains a destructive disease despite the advent of new and aggressive therapeutics. It is widely accepted that environmental and dietary factors play a role in determining one's risk of breast cancer. There is an extensive and growing amount of work devoted to the possible links between diet and a reduction in the risk of breast cancer. Our lab has studied the effects of dietary exposure to genistein, the primary isoflavone component of soybeans. We have shown that genistein administered in neonatal, prepubertal, and a combination of neonatal and prepubertal periods followed by adult exposures can suppress chemically-induced mammary cancer in Sprague-Dawley rats [2-4]. Other dietary compounds that have received much attention for their health benefits, including anti-carcinogenic properties, are the naturally occurring polyphenols resveratrol and EGCG.

Resveratrol is a polyphenolic phytoalexin present in grape skins and red wine that has been shown to have antioxidant and anti-inflammatory properties. In 1996, Jang et al. reported that resveratrol could inhibit a number of cellular events associated with the initiation, promotion, and progression of cancer [5]. That report has been followed with a battery of *in vitro *investigations into the chemopreventive activity of resveratrol [6,7]. Also, there have been reports in which resveratrol has been shown to reduce the induction of chemically-induced breast cancer models. Bhat et al. administered resveratrol *via *gavage and showed an increase in tumor latency and a decrease in the total number of tumors in an N-methyl-N-nitrosourea-(NMU) induced mammary cancer model [8]. Dietary resveratrol also reduced incidence and multiplicity and extended the latency period in a DMBA-induced mammary cancer model [9], although this study used a small number of animals and what the authors called a DMBA dose "suboptimal to produce sufficient tumors". Our studyused dietary administration and employed at least 30 animals per group and a dose of DMBA that was sufficient to cause 100% tumor incidence and resulted in an average of 8.5 tumors per rat in the control group.

The consumption of tea has been associated with a host of health benefits including the prevention of cancer [10]. The chemopreventive effects of tea have been attributed to the large amount of polyphenolic catechins present in tea. A cup of tea contains 30–40% catechins by dry weight, with EGCG being the most prevalent catechin [11]. These catechins are strong antioxidants, have been associated with a reduced risk of cardiovascular disease, and reported to have anti-carcinogenic effects on skin, lung, oral cavity, stomach, colon, pancreas, and breast cancers in animal models [10]. Gupta et al. showed green tea polyphenols can inhibit prostate tumors in a transgenic mouse model [12].

To our knowledge, this is the first study that simultaneously investigated the potential of purified resveratrol and chemically-synthesized EGCG throughout life in the diet to suppress chemically-induced mammary cancer initiated with concentrations of DMBA that result in adenocarcinomas. We also investigated the potential of resveratrol or EGCG *via *the diet to modulate mammary gland maturation, cell proliferation, and apoptosis in terminal mammary structures as mechanisms for cancer chemoprevention.

## Methods

### Animals

This study was approved by the University of Alabama at Birmingham Animal Use Committee. Female Sprague-Dawley CD rats (Charles River, Raleigh, NC) were housed in a climate controlled room with a 12 hour light/dark cycle (light on at 8:00 AM) in the UAB Animal Resources Facility and fed phytoestrogen-free AIN-76A diet (Harlan Teklad, Madison, WI). At birth, litters were placed on one of three diets: 1) AIN-76A with tap water as the control, 2) 1 gram resveratrol/kg AIN-76A dietwith tap water, or 3) 0.065% EGCG in the drinking water with AIN-76A diet. Resveratrol (Xi'an Sino-Dragon Import & Export Co., China) was extracted from Rhizoma Polygoni Cuspidati and tested as 98% pure by HPLC. EGCG (a gift from Roche Fine Chemicals, Basel, Switzerland) was chemically-synthesized at 93% purity as tested by HPLC. The bottles used to administer all drinking fluids were an amber color to suppress EGCG degradation. EGCG was changed daily around 3 PM. Treatments were started at birth and continued throughout the life of the animals, with the rats having free access to both food and drink. The resveratrol and EGCG doses were chosen based on previous work by Bhat et al. and Gupta et al. [8,12]. Bhat reported an inhibition of carcinogen-induced mammary tumors by gavaging rats with 100 mg resveratrol/kg body weight. Hence, we calculated that a 200 gram rat eating 20 grams of the provided diet with 1 mg resveratrol/g diet would consume ~100 mg resveratrol/kg body weight. Gupta showed a reduction in prostate tumors in the transgenic adenocarcinoma of the mouse prostate (TRAMP) model using a 0.1% solution of green tea polyphenols (62% of which was EGCG). Animals were weaned at 21 days postpartum. Body weights and food and drink consumptions were measured at 21 and 50 days postpartum. Onset of vaginal opening was recorded as a marker for sexual maturity.

### Tumor induction

At 50 days postpartum, 94 female rats (30 Control, 30 Re sveratrol, and 34 EGCG) were gavaged with 60 mg dimethylbenz[a]anthracene (DMBA)/kg body weight, a dose sufficient to cause 100% tumor incidence in the control group over the course of the study. The DMBA was dissolved in sesame oil at a stock solution of 30 mg/ml. Animals were palpated twice a week starting five weeks after DMBA administration in order to record the presence, location, size, and date of detection for all tumors. Animals were sacrificed when the tumor diameter reached one inch, animals became moribund, or rats reached 18 weeks post-DMBA treatment. Sections from the tumors were fixed in 10% neutral buffered formalin and embedded in paraffin. Tumor blocks were sectioned and fixed on slides to be evaluated histopathologically by Dr. I. Eltoum, a board certified pathologist at UAB.

### Mammary gland differentiation

Whole mounts of the fourth abdominal mammary glands were prepared from 50-day-old rats as previously described [2]. Briefly, mammary glands were removed at time of sacrifice and placed on a microscope slide. Glands were fixed in neutral buffered formalin followed by de-fatting in acetone. The glands were stained using 0.2% alum carmine (Sigma, St. Louis, MO). Slides were dehydrated in a series of graded alcohols from 35–100%. The slides were cleared in xylene and compressed between two glass slides. The glands were allowed to expand before being mounted using glass coverslips. Glands were analyzed using a Nikon light microscope (Nikon, Melville, NY) for the quantity of terminal ductal structures including: end buds, ducts, and lobules types I and II as previously described [2,3,13]. A terminal end bud was characterized as an elongated ductal structure with 3 to 6 epithelial cell layers and >100 micrometers in diameter. Terminal ducts have one to three epithelial cell layers and were <100 micrometers in diameter, while lobules I and II have 5–10 and 10–20 alveolar buds, respectively.

### Cell proliferation

Bromodeoxyuridine (BrdU) (Sigma) incorporation was used as an index of cell proliferation. BrdU was administered two hours before sacrifice, injected i.p. with 100 mg BrdU/kg body weight. The contralateral fourth mammary gland was removed and fixed in 10% neutral buffered formalin. The glands were then placed in 70% ethanol overnight before being embedded in paraffin. The blocks were sectioned onto microscope slides. Slides were analyzed as previously done in our lab [3]. Briefly, paraffin was removed by xylene and the slides were rehydrated in ethanol. Slides were placed in 3.5 N HCl and then trypsinized. Slides were placed in 3% H_2_O_2 _(Sigma) to quench endogenous peroxidase and blocked in 10% normal horse serum (Vector, Burlingame, CA). Slides were incubated with monoclonal anti-BrdU primary antibody (Sigma) and subsequently incubated with biotinylated horse anti-mouse secondary antibody (Vector). Detection was performed using streptavidin (ABC reagent, Vector) and the color developed by DAB (Vector). Slides were counterstained with Gills no. 2 hematoxylin and then dehydrated, cleared, and coverslipped. The labeling index, which is the number of cells incorporating BrdU dividedby total number of cells × 100, was determined for terminal end buds, terminal ducts, and lobules types I and II using a Nikon light microscope and Nikon digital camera, and analyzed using Image J software (NIH).

### Apoptosis assay

The TdT-FragEL™ DNA Fragmentation Detection Kit (Calbiochem, San Diego, CA) was used to measure apoptosis following the manufacturer's instructions. Briefly, paraffin-embedded tissue sections were deparaffinized and rehydrated in graded alcohols. Tissues were permeablized with Proteinase K and rinsed with Tris-Buffered Saline. Endogenous peroxidases were inactivated with 3% hydrogen peroxide and labeled with TdT enzyme. The labeling reaction was terminated with Stop Buffer. The tissues were blocked and then color detection was done with diaminobenzidine (DAB). The slides were couterstained with methyl green, dehydrated in alcohol and xylene, and coverslipped. The apoptotic index was the number of epithelial cells stained positive for apoptosis divided by the total number of epithelial cells counted in both mammary terminal end buds and lobule structures. Visualization was performed using a Nikon light microscope, Nikon digital camera, and analyzed using Image J software (NIH).

### Serum polyphenol concentrations

Blood was collected from 50 day old rats at the time of sacrifice. Whole blood was spun at 2300 rpm for 15 minutes to collect serum and frozen at -80°C until use. Resveratrol and EGCG were extracted from the serum and measured on a 4000 Q TRAP^® ^LC/MS/MS System (Applied Biosystems, Foster City, CA). Serum extractions were done as previously described [[3] and [14]]. For resveratrol, internal standards (apiginin, 4-methylumbelliferone, and phenolphthalein glucuronide) were added to serum followed by incubation with 1 mg β glucuronidase/sulfate enzyme (Sigma) in 250 uL ammonium acetate buffer. Samples were extracted in hexane and followed by ether extraction. Samples were redissolved in 80% methanol prior to LC/MS/MS analysis. For EGCG serum analysis, 1% acetic acid in 100% methanol was used after incubation with internal standards and β-glucuronidase enzyme. A separate standard curve was run for each experiment. Control serum was used as a negative control for both resveratrol and EGCG extractions.

### Serum concentrations of 17β-estradiol, progesterone, and prolactin

Serum estradiol-17β, progesterone, and prolactin concentrations were measured using radio-immunoassays (Diagnostic Systems Laboratories, Webster, TX) as described by the manufacturer. All samples were run in duplicate.

### Statistics

The time-to-event data, e.g., time-to-first-tumor (latency) and time-to-sacrifice (tumor burden), were analyzed using the LIFETEST and LIFEREG procedures in SAS^® ^[15]. Survival functions were first estimated nonparametrically using Kaplan-Meier (KM) and compared across the three groups using the log-rank test and parametrically using Weibull regression analysis. Those animals that did not demonstrate the event in question by the end of study or before sacrifice were treated as censored in the time-to-event analysis and the end of study or sacrifice time substituted in for their actual times. Tumor multiplicity data were analyzed with the GENMOD (generalized linear models) procedure in SAS using generalized Poisson regression on the tumor appearance rates (assuming a negative binomial distribution). The rate of tumor appearance was computed for each animal as the total number of tumors divided by the number of days on the study. For more detail about generalized linear models and associated maximum likelihood estimation, see McCullagh and Nelder [16]. All biochemical measurements were done using Students t test with a p value of 0.05 as statistically significant, while group comparisons were analyzed by one way analysis of variance (ANOVA) in SAS.

## Results

### Body and uterine weights, and vaginal opening

Lifetime treatments with 1 g resveratrol/kg diet or 0.065% EGCG in the drinking water did not result in a significant difference in body weights as assessed at 21 or 50 days postpartum (Table 1). Likewise, there was no difference in the uterine/body weight ratio between the groups at ages 21 or 50 days. The average number of days to vaginal opening, a marker for sexual maturation, was not altered by resveratrol (34.95 ± 0.35) or EGCG (35.04 ± 0.35) treatment as compared to the control rats (34.16 ± 0.40) There were no observed differences in food or drink consumption between the control and either polyphenol treatment in 21, 35, 50, or 100 day old rats (data not shown).

**Table 1:** Body weights and uterine to body weight ratios for female rats exposed to resveratrol or EGCG.

**Treatment Age**	**Body Weight (g)**	**Uterine: BW Ratio**
**Control 21 Day**	53.30 ± 1.09	0.57 ± 0.02
**Resveratrol 21 Day**	53.45 ± 1.25	0.56 ± 0.03
**EGCG 21 Day**	53.80 ± 0.67	0.53 ± 0.03
**Control 50 Day**	186.29 ± 3.25	1.79 ± 0.12
**Resveratrol 50 Day**	183.45 ± 2.84	1.95 ± 0.10
**EGCG 50 Day**	190.80 ± 2.63	1.54 ± 0.08

Female offspring of Sprague-Dawley CD rats were exposed to resveratrol (1 g/kg AIN-76A diet), EGCG (0.065% in drinking water), or AIN-76A alone as the control diet from birth to either 21 or 50 days postpartum. Values represent means ± SEM.

### Tumor studies

**Figure 1:**
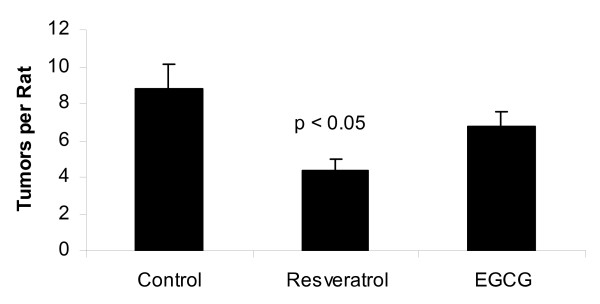
**Tumor multiplicity in female Sprague-Dawley CD rats exposed to resveratrol, EGCG, or AIN-76A (control) from birth until sacrifice**. On day 50 postpartum, all animals were treated with 60 mg DMBA/kg body weight. Figure 1 shows tumor multiplicity, with the values representing mean tumors per rat ± 2 standard errors. A p value < 0.05 was considered statistically significant.

**Figure 2:**
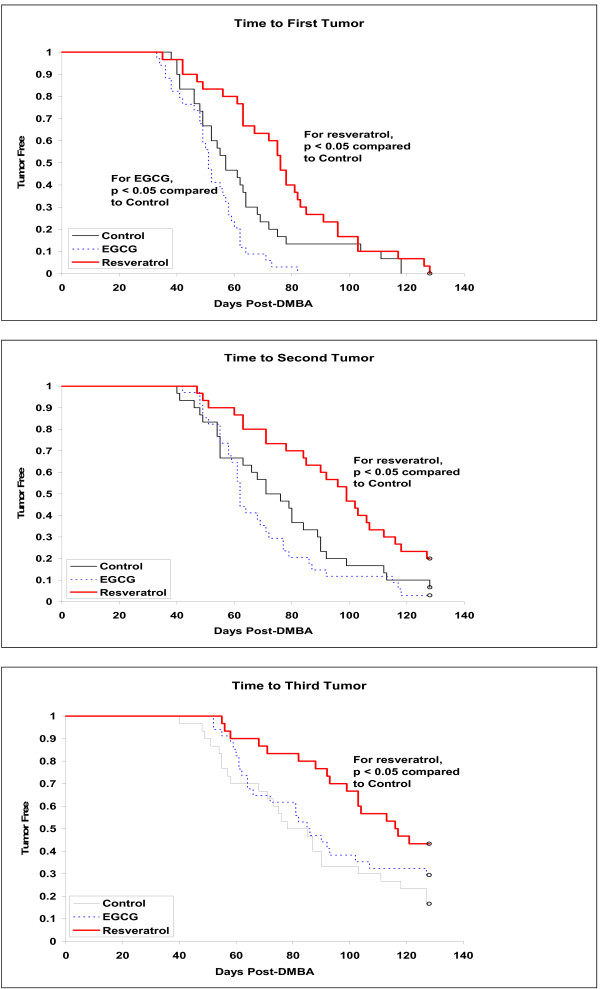
**Tumor latency (2A, B, C) in female Sprague-Dawley CD rats exposed to resveratrol, EGCG, or AIN-76A (control) from birth until sacrifice**. On day 50 postpartum, all animals were treated with 60 mg DMBA/kg body weight. Figures 2A, 2B, and 2C depict the time to first tumor, second tumor, and third tumor (latency), respectively. A p value < 0.05 was considered statistically significant.

Female Sprague-Dawley rats exposed to resveratrol in the diet throughout life and to DMBA on day 50 postpartum had significantly lower tumor multiplicity rates (Figure 1) and significantly longer tumor latency (Figure 2A, B, and 2C) compared to control rats receiving DMBA. Animals exposed throughout life to EGCG in the drinking water showed a decrease in the latency to first tumor development, although there was no significant difference as compared to the control group with respect to second and third tumor latency (Table 2A, B, and 2C). Likewise, tumor multiplicity in EGCG-exposed rats was not significantly different from the controls. Animals receiving resveratrol developed the lowest number of chemically-induced mammary tumors per rat (4.39 ± 0.61) (Figure 1). This was a 50% reduction in the number of mammary tumors compared to control animals (8.79 ± 1.33; p < 0.001). At the time of necropsy, animals that received EGCG in the drinking water had fewer mammary tumors per rat than the controls (6.74 ± 0.81 as compared to 8.79 ± 1.33), but this did not reach statistical significance (p = 0.126) (Figure 1). Both resveratrol and EGCG had a significant influence on the latency of tumor development (Table 2 and Figure 2A, B, and 2C). Rats exposed to resveratrol had a significantly delayed onset of the first mammary tumor (76 days) as compared to the control animals (57 days; p < 0.05). Animals exposed to EGCG had a slight, but significantly enhanced onset of the first mammary tumor (51 days) compared to control rats (57 days; p < 0.05). This trend of delayed time to tumor development continued for the second and third times to tumor development in the resveratrol-treated rats, while the animals treated with EGCG were not significantly different than the control group (Table 2). Neither EGCG nor resveratrol had an effect on mammary tumor incidence as almost every animal (93 out of 94) developed at least one tumor by the end of the study. One resveratrol-treated animal did not develop any tumors. All histologically evaluated tumors were found to be adenocarcinomas. These results demonstrated that treatment of resveratrol in the diet significantly reduced the number and increased the time to onset of DMBA-induced mammary tumors. EGCG treatment showed no significant difference in the number of mammary tumors per rat compared to the control animals.

**Table 2:** Tumor latency to first, second and third tumor development.

**Treatment**	**Median Time to First Tumor**	**p value**
Control	57 days	-
Resveratrol	76 days	0.029
EGCG	51 days	0.017

**Treatment**	**Median Time to Second Tumor**	**p value**

Control	73.5 days	-
Resveratrol	99 days	0.014
EGCG	62 days	0.239

**Treatment**	**Median Time to Third Tumor**	**p value**

Control	81.5 days	-
Resveratrol	116.5 days	0.009
EGCG	85.5 days	0.309

Female offspring of Sprague-Dawley CD rats were exposed to resveratrol (1 g/kg AIN-76A diet), EGCG (0.065% in drinking water), or AIN-76A alone as the control diet starting at birth and continued throughout life. Each group contained a minimum of 30 rats. DMBA was administered at 60 mg/kg body weight at 50 days postpartum. The rats were palpated for tumors twice per week and the day of each tumor appearance was recorded. All p values are measured against the control group.

### Mammary gland differentiation, cell proliferation, and apoptosis

The predominant mammary terminal ductal structures found in 50-day-old control rats were terminal end buds (37%), terminal ducts (31%), lobules type I (23%), and lobules type II (9%). While resveratrol in the diet did not significantly alter the numbers of individual structures, the combined lobules types I and II were significantly increased compared to the control group (Table 3). On the other hand, EGCG in the diet significantly decreased the number of terminal ducts but did not affect terminal end buds, lobules I or lobules II.

**Table 3:** Terminal ductal structures in mammary glands of female rats exposed to resveratrol or EGCG in the diet.

**Treatment**	**Terminal Ducts**	**Terminal End Buds**	**Lobules Type I**	**Lobules Type II**	**Lobules Type I and II**
**Control**	44 ± 5	53 ± 3	32 ± 3	13 ± 2	44 ± 4
**Resveratrol**	41 ± 6	58 ± 3	42 ± 3	15 ± 3	59 ± 5^A^
**EGCG**	28 ± 3^A^	39 ± 9	34 ± 4	16 ± 2	50 ± 4

Female offspring of Sprague-Dawley CD rats were exposed to resveratrol at 1 g/kg AIN-76A diet, 0.065% EGCG in the drinking water, or AIN-76A diet alone from birth to 50 days postpartum. The fourth abdominal mammary gland was measured in a minimum of seven rats per group. Values for terminal ductal structures represent means ± SEM. ^A ^represents a p value < 0.05. The p value is compared to the control group.

BrdU incorporation was used as an index of cell proliferation. In the mammary glands of 50-day-old control rats, the labeling indices were ~5% in terminal end buds and terminal ducts and ~2% in lobules type I. Treatment with resveratrol in the diet significantly reduced cell proliferation in all of the terminal ductal structures that were measured (terminal ducts, terminal end buds, and lobules I) (Table 4). EGCG treatment did not alter the proliferation indices for any of the mammary terminal ductal structures as compared to the control group.

**Table 4:** Cell proliferation in mammary terminal ductal structures of 50-day-old rats exposed to resveratrol or EGCG.

**Treatment**	**Terminal End Buds**	**Terminal Ducts**	**Lobules**
**Control**	4.83 ± 0.61	5.03 ± 1.07	1.75 ± 0.22
**Resveratrol**	2.50 ± 0.75^A^	0.79 ± 0.27^A^	0.90 ± 0.14^A^
**ECGC**	4.56 ± 0.31	4.54 ± 0.97	2.15 ± 0.31

Female Sprague-Dawley CD rats were exposed from birth to 50 days postpartum to: resveratrol (1 g/kg diet), EGCG (0.065% in water), or AIN-76A as the control. Two hours prior to sacrifice the rats were injected with 100 mg BrdU/kg body weight. The labeling index was determined in three of each type of terminal structure (terminal ducts, terminal end buds, lobules) per mammary gland. A minimum of 7 rats was used in each group. Values represent means ± SEM. ^A ^represents a p value < 0.05.

A DNA fragmentation assay was used to measure apoptosis. An apoptotic index was determined for mammary terminal end buds and lobules (combined type I and II) from 50-day-old rats. Rats treated with resveratrol showed a significant increase in apoptotic index in the mammary epithelial cells in terminal end buds as compared to the control rats (Table 5). EGCG in the diet did not have an effect on apoptosis in the epithelial cells of terminal end buds as compared to the controls. Neither treatment affected the apoptotic index in mammary lobules.

**Table 5:** Apoptotic index for epithelial cells in mammary terminal ductal structures of rats 50 days postpartum exposed to resveratrol or EGCG.

**Treatment**	**Terminal End Buds**	**Lobules**
**Control**	35.43 ± 1.37	39.39 ± 2.67
**Resveratrol**	44.18 ± 1.84^A^	41.24 ± 2.38
**Control**	46.10 ± 1.82	43.91 ± 1.97
**EGCG**	49.31 ± 1.57	43.80 ± 2.52

Female Sprague-Dawley rats were exposed to resveratrol or EGCG in the diet from birth until 50 days postpartum. Apoptotic indices (positively stained cells/total cells) were determined from mammary epithelial cells from terminal end buds and lobule structures, with at least 7 animals per group. Values represent mean ± SEM. ^A ^represents a p value < 0.05.

### Serum polyphenol, 17β-estradiol, progesterone, and prolactin concentrations

Resveratrol and EGCG concentrations were measured in the serum of 50-day-old animals. In the serum of rats exposed to resveratrol in the diet, total resveratrol concentration was 2.00 ± 0.64 μM. In the serum of the EGCG treated group, at 50 days of age, the average concentration of total EGCG was 31.06 ± 3.05 nM. All rats on the control diet (AIN-76A) had non-measurable serum concentrations of resveratrol or EGCG.

The concentrations of 17β-estradiol, progesterone, and prolactin were measured in the serum of 50-day-old rats treated with resveratrol or EGCG in the diet or drinking water respectively (Table 6). There were no statistical differences in serum estradiol levels between control (11.39 ± 4.56 pg/ml), resveratrol-treated (13.87 ± 2.28 pg/ml), or EGCG-treated rats (5.90 ± 1.64 pg/ml). Likewise, serum progesterone concentrations were not altered by resveratrol (14.98 ± 2.73 ng/ml) or EGCG treatment (9.64 ± 1.45 ng/ml) as compared to the control rats (12.42 ± 2.70 ng/ml). Neither resveratrol nor EGCG treatment affected serum prolactin concentrations as compared to the controls (1.24 ± 0.06, 1.19 ± 0.04, and 1.25 ± 0.10 respectively).

**Table 6:** Serum concentrations of 17β-estradiol, progesterone, and prolactin in 50-day-old female rats, exposed to resveratrol or EGCG.

**Treatment**	**17β-Estradiol (pg/ml)**	**Progesterone (ng/ml)**	**Prolactin (ng/ml)**
**Control**	11.39 ± 4.56	12.42 ± 2.70	1.25 ± 0.10
**Resveratrol**	13.87 ± 2.28	14.98 ± 2.73	1.19 ± 0.04
**EGCG**	5.90 ± 1.64	9.64 ± 1.45	1.24 ± 0.06

Female Sprague-Dawley CD rats were exposed from birth to 50 days postpartum to: resveratrol (1 g/kg diet), EGCG (0.065% in water), or AIN-76A as the control. Blood was collected from female rats determined to be in the estrous phase of the estrous cycle (8/group). 17β-estradiol, progesterone, and prolactin concentrations were measured by radioimmunoassay. Values represent the mean ± SEM.

## Discussion

### Body and uterine weights and vaginal opening

Resveratrol and EGCG given throughout life at concentrations of 1 g resveratrol/kg diet and 65 mg EGCG/100 mL water, respectively did not cause any significant alterations on body or uterine weights in 21- and 50-day-old female Sprague-Dawley rats. Dietary treatment throughout life encompasses lactational exposure until time of weaning at day 21 postpartum, plus eating and drinking on their own after day 14. The number of days until vaginal opening, a marker for sexual maturation, was measured with no significant difference between any of the groups. Resveratrol and EGCG at the given concentrations did not cause toxicity as observed by loss of weight or differences in food and drink consumption. The lack of alteration of body weights was expected as Juan *et al*. showed that daily administration of resveratrol (20 mg/kg) had no effect on final body weights or on the tissue weights of the lungs, heart, liver, kidney, or adrenal glands [17]. Likewise, Hirose *et al*. showed no differences in body, liver, or kidney weights after treatment with green tea catechins in the diet or in the water [18].

### Chemoprevention

Resveratrol given throughout life in the diet resulted in a suppression of DMBA-induced mammary cancer. Rats exposed to resveratrol had significantly fewer tumors per animal compared to those that were not exposed to resveratrol, and the latency (time to first, second and third tumors) was also significantly extended in the animals exposed to resveratrol. The effect of increasing tumor latency became more pronounced with each tumor. The chemoprotective effect of resveratrol on the mammary gland supports previous reports that resveratrol can suppress chemically-induced mammary carcinogenesis. Bhat *et al*. showed protection with resveratrol *via *gavage against NMU-induced mammary cancer, while Banerjee *et al*. showed protection against DMBA-induced mammary tumorigenesis [8,9], although the latter study was conducted with a small number of animals and used a suboptimal dose of DMBA which resulted in less than 80% tumor incidence in the control group and fewer than 2.5 tumors per rat. We chose to use a statistically more robust 30 animals per group and a dose of DMBA that resulted in 100% incidence of mammary adenocarcinomas in the control group over the course of the experiment, with an average of more than 8 tumors per rat in the control group. All tumors examined histologically were determined to be adenocarcinomas.

Exposure to 0.065% EGCG in the drinking water, throughout life, failed to protect the mammary gland against DMBA-induced mammary cancer. As mentioned above, the dose of EGCG was equivalent to one that inhibited prostate tumors in the TRAMP model with green tea polyphenol extract [12]. The tumor multiplicity in the EGCG-treated group was not significantly different from the animals that did not receive EGCG. While the time-to-first tumor latency was slightly (yet significantly) shorter than that of the control animals, the times-to-second and -third tumors did not differ from that of the controls. We interrupt this to mean that pure EGCG, at the given dose, did not increase or decrease chemically-induced mammary cancer. Not finding a chemopreventive effect using EGCG is in line with several reports that show EGCG and green tea as having minimal effects on mammary carcinogenesis. Hirose *et al*. showed that a green tea polyphenol fraction had no effect on tumor incidence or multiplicity in a DMBA-induced mammary cancer model [18,19]. This lack of effect could be due to several factors, including the low reported bioavailability of EGCG [20]. Interestingly, Chen *et al*. reported that rats receiving decaffeinated green tea displayed a higher plasma concentration of EGCG than rats receiving pure EGCG, even though the dose of pure EGCG was five times higher [21]. This could implicate the other catechins found in green tea as playing a role in the bioavailability of EGCG and possibly the mammary-protective effects using whole green tea polyphenols or a mixture of green tea catechins [22,23]. It is important to study whether EGCG is the most active player in green tea or whether it is the mixture of catechins that is important for the health related effects.

### Mammary gland differentiation

The effects of resveratrol and EGCG as given through the diet and drinking water, respectively were evident by the changes in the number and distribution of the types of mammary terminal ductal structures. At 50 days post-partum, the time of carcinogen exposure, there was no statistical difference in the numbers of terminal end buds between any of the groups. Terminal end buds are the least differentiated terminal ductal structures in the mammary gland and reported to be the most susceptible to carcinogen exposure [13]. Reduction in the number of terminal end buds has been correlated with a reduction in tumor multiplicity in rats exposed prepubertally to the phytoestrogen genistein [2-4]. The animals exposed to resveratrol throughout life had slightly, though significantly, more combined lobule structures at 50 days post-partum. Lobules types I and II are the most differentiated structures and this increase in gland maturation can help to explain the protective effects seen with exposure to resveratrol *via *the diet. Chemoprevention experiments in our lab with the phytoestrogen genistein, a component of soy, have shown a reduction in mammary tumor multiplicity correlated with fewer terminal end buds and increased lobules at 50 days postpartum [2-4]. Hilakivi-Clarke *et al*. also showed that the mammary glands of rats treated with high n-3 polyunsaturated fatty acids have more lobules and are less susceptible to carcinogen insult [24]. Thus, enhanced maturation of the mammary gland can protect against carcinogen insult. EGCG-treated animals showed no increase in lobule structures and no decrease in the number of terminal end buds, which could influence the lack of protection against carcinogenesis.

### Cell proliferation

Cellular proliferation in susceptible tissue structures is extremely important in the oncogenic-response to carcinogens. Russo *et al*. have shown a greater oncogenic response in active, dividing cells in the mammary gland of rats, with the active cells in the terminal end buds being the most responsible for subsequent, DMBA-induced adenocarcinomas [13,25-27]. In our work, at 50 days postpartum, resveratrol-treated rats showed a significant reduction in the percentage of proliferating cells in the mammary terminal end buds as well as in terminal ducts and lobules. This reduction in proliferative cells in the terminal ductal structures of the mammary gland could contribute to the protective effect against mammary carcinogenesis. On the other hand, the labeling index for EGCG-treated rats in all terminal ductal structures was not statistically different than the control rats. No reduction of cell proliferation at 50 days postpartum could help to explain the lack of mammary cancer suppression observed with EGCG as compared to resveratrol.

### Apoptosis

The apoptotic labeling index for epithelial cells in mammary terminal end buds in the resveratrol-treated animals was increased 25% compared to the control rats. Though a modest increase, the increase in apoptosis reached statistical significance. The rats treated with EGCG showed no statistical difference in apoptotic index in the mammary terminal end buds compared the control animals. Neither treatment affected the apoptotic index for mammary lobular structures. Resveratrol has been reported to induce apoptosis in a number of cancer cell lines, including breast carcinoma cell lines [reviewed in [6]]. This increase in mammary epithelial cell apoptosis in the terminal end buds after resveratrol treatment, coupled with the significant reduction in cell proliferation could create an environment in the mammary gland that is less susceptible to chemical carcinogenesis.

### Serum polyphenol concentrations

Bioavailability is an important consideration when dealing with chemopreventive agents that are consumed in the diet. There are multiple reports that have measured the serum polyphenol concentrations in humans and rodents of both resveratrol and EGCG. At the present dose of resveratrol given in the diet, 1 g/kg diet, a 200 gram rat that consumed 20 grams of diet would receive ~100 mg resveratrol/kg body weight. The total resveratrol concentration measured in the serum of these resveratrol treated rats was 2.00 ± 0.64 μM. Crowell *et al*. administered 300 mg resveratrol/kg body weight/day by gavage and reported a serum concentration of 1.46 μM [28]. Resveratrol treatment, administered via gavage to rats at 20 and 50 mg/kg body weight respectively [29,30], gave maximum serum concentrations in the low micromolar range, consistent with our results. Our results were also similar to the total serum concentrations of resveratrol in humans (12 healthy males) receiving 0.36 mg resveratrol/kg body weight dissolved in white wine [31]. This dose is about 20 times higher than the amount of resveratrol one could receive after moderate wine consumption. Interestingly, Gescher and Steward point out in a commentary that low doses of resveratrol, and thus low resveratrol serum concentrations, may suffice to exert a potent chemopreventive effect [32]. Further studies are necessary to discern the effects of lower doses of resveratrol on mammary carcinogenesis.

In our study with EGCG, female rats received 0.065% EGCG in the drinking water, made fresh each afternoon. Thus, a 200 g female consuming 25 mL of fluid would receive 16.25 mg EGCG per day (81 mg EGCG/kg body weight). Again, this dose was extrapolated from the work of Gupta *et al*. who showed that a 0.1% enriched fraction of green tea polyphenols (62% EGCG) could inhibit prostate cancer in the TRAMP mouse model [12]. That report also stated that the dose "mimics an approximate consumption of six cups of green tea per day by an average adult human." At the dose administered, the total EGCG concentration found in the serum was 31.06 ± 3.05 nM. This is similar to the work of Chen *et al*. who treated male rats with 75 mg EGCG/kg body weight by gavage and reported a serum concentration of total EGCG at 43 nM [21]. Several human studies have employed doses of EGCG that would be equivalent to ~2–3 cups of green tea for an average human. Lee *et al*. administered 20 mg green tea solids/kg body weight and reported a maximum serum EGCG concentration of 172 nM [33]. Another group using a similar dose reported a maximum serum concentration of total EGCG at ~720 nM [34]. Species differences could account for the lower serum concentrations of EGCG observed in the rat. It has been reported that most of the EGCG does not get into the blood, but is excreted through the bile to the colon [21]. Also, higher serum concentrations of EGCG have been reported when green tea polyphenol mixtures have been administered as opposed to pure EGCG [21]. The low circulating polyphenol concentration of EGCG could play a role in the lack of effect observed on tumor multiplicity and latency observed in this study.

### Serum 17β-estradiol, progesterone, and prolactin concentrations

At 50 days postpartum, resveratrol in the diet or EGCG in the drinking water did not significantly influence the serum concentrations of estradiol, progesterone, or prolactin. The serum concentrations of estradiol for control, resveratrol-, and EGCG-treated animals were 11.4, 13.9, and 5.9 pg/ml, respectively. Lamartiniere et al. have reported similar serum estradiol concentrations (~10–12 pg/ml) after dietary treatment with AIN-76A or 250 mg daidzein/kg diet [35]. The same report showed serum progesterone concentrations in the low ng/ml range, very similar to what we saw with serum progesterone concentrations after resveratrol or EGCG treatment. Though not reaching statistical significance, EGCG tended to lower serum estradiol concentrations as compared to the control rats (5.90 ± 1.64 versus 11.39 ± 4.56 nM). This is in line with the previous reports of Kao *et al*. [36] and Hangata *et al*. [37] who showed in rats and humans, respectively that green tea treatment could lower serum estradiol concentrations. Serum prolactin was not significantly altered by treatment with resveratrol or EGCG. This is consistent with Okazaki *et al*., who reported no difference in serum prolactin levels after exposure to the polyphenol genistein [38].

### Summary

Our work supports the previous reports that resveratrol in the diet is effective at inhibiting DMBA-induced mammary cancer. We have shown that resveratrol can enhance maturation of the mammary gland as well as reduce cellular proliferation and increase apoptosis in mammary epithelial cells, in a manner that is protective against mammary carcinogenesis. Coupling this with our previous work with genistein [2-4], we plan to use a combination of genistein and resveratrol in the diet to determine whether there is an additive or synergistic effect of these natural polyphenols to suppress mammary carcinogenesis. As for the administration of EGCG in the drinking water, no mammary protective effect was demonstrated. It remains to be seen whether a higher dose, different route of administration, a different tea catechin, or a mixture of green tea catechins can protect against mammary cancer.

There is still much that remains unknown about the *in vivo *molecular mechanisms of resveratrol and EGCG that play a role in their effects on mammary carcinogenesis. The effects on estrogen receptor pathways as well as other steroid and growth factor pathways and effects on enzymes that are important in the activation or removal of xenobiotics must be elucidated such that these polyphenols can be recommended for clinical trials and help in the prevention of breast cancer.

## Authors' contributions

TGW carried out the carcinogenesis study, whole mount analysis, cell proliferation and apoptosis assays, and drafted the manuscript. MC performed the statistical analysis for tumor multiplicity and tumor latency, wrote the statistical methods, and assisted in writing the manuscript. CAL proposed the study design and assisted in writing the manuscript.
